# Evaluation of the QIAstat-Dx Meningitis/Encephalitis Panel, a multiplex PCR platform for the detection of community-acquired meningoencephalitis

**DOI:** 10.1128/jcm.00426-23

**Published:** 2023-09-13

**Authors:** Thomas Sundelin, Johanna Bialas, Juana de Diego, Markus Hermanowski, Hendrik Leibhan, Léa Ponderand, Marti Juanola-Falgarona, Tara Jones, Melisa Rey, Sarah Johnson, Josep Pareja, Yvan Caspar

**Affiliations:** 1 Department of Clinical Microbiology, Herlev and Gentofte Hospital, Herlev, Denmark; 2 Labor Berlin-Charite Vivantes Services GmbH, Berlin, Germany; 3 Laboratoire de bactériologie, CHU Grenoble Alpes, La Tronche, France; 4 Université Grenoble Alpes, CEA, CNRS, IBS, Grenoble, France; 5 STAT-Dx Life S.L. (a QIAGEN Company), Carrer de Baldiri Reixac, Barcelona, Spain; 6 QIAGEN Ltd, Manchester, United Kingdom; NorthShore University HealthSystem, Evanston, Illinois, USA

**Keywords:** meningitis, encephalitis, QIAstat-Dx, multiplex, real-time PCR, molecular diagnostics

## Abstract

Rapid identification of the causative pathogens of central nervous system infections is essential for providing appropriate management and improving patient outcomes. The performance of QIAstat-Dx Meningitis/Encephalitis (ME) Panel—a multiplex PCR testing platform—in detecting pathogens implicated in meningitis and/or encephalitis was evaluated using BioFire FilmArray ME Panel as a comparator method. This multicenter study analyzed 585 retrospective residual cerebrospinal fluid specimens and 367 contrived specimens. The QIAstat-Dx ME Panel showed positive percent agreement (PPA) values of 100% for *Neisseria meningitidis*, *Streptococcus agalactiae*, *Escherichia coli* K1, *Listeria monocytogenes*, and *Cryptococcus gattii*/*neoformans* on clinical samples compared to the BioFire FilmArray ME Panel. The PPA values observed for *Haemophilus influenzae* and *Streptococcus pneumoniae* were 80% and 88.24%, respectively. Negative percent agreement (NPA) values were >99.0% for each of the six bacterial targets and one fungal target tested with clinical samples. One viral target, herpes simplex virus 1, exhibited a PPA value of 100.0%, while the remaining viral targets—human parechovirus, herpes simplex virus 2, human herpes virus 6, and varicella zoster virus—were >90.0%, with the exception of enterovirus, which had a PPA value of 77.8%. The QIAstat-Dx ME Panel detected five true-positive and four true-negative cases compared to BioFire FilmArray ME Panel. The NPA values for all viral pathogens were >99.0%. Overall, the QIAstat-Dx ME Panel showed comparable performance to the BioFire FilmArray ME Panel as a rapid diagnostic tool for community-acquired meningitis and encephalitis.

## INTRODUCTION

Traditional methods for diagnosing meningitis and encephalitis in clinical settings are neither sufficiently sensitive nor timely. Clinical presentation is complicated by the frequent presentation of nonspecific and overlapping symptoms (e.g., headache, fever, altered mental status), potentially leading to delays in therapy and significant clinical consequences (https://www.who.int/health-topics/meningitis). The etiology remains unidentified in about 5% of encephalitis cases and as many as 60% of meningitis cases (1), underscoring the need for easy-to-use diagnostic modalities that can rapidly detect and identify the causative agent in order to improve patients’ clinical outcomes. Diagnostic algorithms can help differentiate between bacterial and viral meningitis (2 – 6), but none are 100% sensitive on validation for bacterial meningitis, potentially leading to missed diagnoses and delayed antibiotic therapy (3). Laboratory-based diagnosis involves analyzing the cellular and chemical parameters of cerebrospinal fluid (CSF) such as protein, glucose levels, and leukocyte count, and performing CSF culture (7). Although it has long been regarded as the gold standard for diagnosing bacterial meningitis, CSF culture has several drawbacks that can impede diagnosis and treatment initiation, including the following: length of time required for bacterial culture (1–5 days); potential for decreased sensitivity in CSF samples from patients taking antibiotics; and difficulty in culturing certain species of bacteria, which can also reduce sensitivity (8, 9).

Advances in PCR diagnostic technologies have been harnessed to enable accurate diagnosis of meningitis and encephalitis by rapidly detecting causative microorganisms in patients’ CSF samples (10). In recent years, multiplex PCR technology has been developed for making this detection even more efficient by rapidly screening for multiple causative pathogens in a single reaction. The QIAstat-Dx Meningitis/Encephalitis (ME) Panel (QIAGEN, Germany), a Conformité Européenne - In-Vitro Diagnostic Devices Directive (CE-IVDD) marked device, is the second panel that is a large multiplex PCR cassette-based panel for diagnosing ME. This assay uses a qualitative closed-system platform with a panel that can simultaneously detect and identify up to 15 different microbes implicated in the pathogenesis of meningitis and/or encephalitis, including eight bacterial, six viral, and one fungal targets in approximately 79 min. These targets include *Escherichia coli* K1, *Haemophilus influenzae*, *Listeria monocytogenes*, *Neisseria meningitidis* (encapsulated), *Streptococcus agalactiae*, *Streptococcus pneumoniae*, *Mycoplasma pneumoniae*, *Streptococcus pyogenes*, herpes simplex virus 1 (HSV-1), herpes simplex virus 2 (HSV-2), human herpes virus 6 (HHV-6), enterovirus, human parechovirus (HPeV), varicella zoster virus (VZV), and *Cryptococcus neoformans*/*gattii*. In addition to qualitative results, cycle threshold (Ct) values and amplification curves are viewable for detected pathogens.

In this study, we evaluated the performance of the QIAstat-Dx ME Panel using the BioFire FilmArray ME Panel as a comparator assay. The multicenter investigation was conducted at three European testing sites using retrospective residual CSF specimens from patients who showed signs and symptoms of meningitis and/or encephalitis.

## MATERIALS AND METHODS

### Retrospective clinical specimens

This investigation was carried out over 6 weeks (October through November 2021) at three different study sites in Europe: Grenoble Alpes University Hospital, La Tronche, France; Labor Berlin-Charité Vivantes Services GmbH, Berlin, Germany; and Herlev Hospital, Herlev, Denmark. CSF specimens enrolled in this investigation fulfilled the following criteria. The CSF specimens were collected by lumbar puncture from both children and adults who exhibited signs and symptoms of meningitis and/or encephalitis. These were residual specimens that had undergone standard of care (SoC) testing, including at least one of the following assays: bacterial culture, PCR, antigen screen, and/or BioFire FilmArray ME Panel. In this study, retrospective frozen samples stored at −80°C for up to 11 years were examined. Each of these specimens had a total volume of 400 µL or more and had not been centrifuged. Specimens were excluded from the study if they had been damaged, lacked clear identification or label, or had been obtained from an external ventricular drain or shunt source. Repeat specimens from the same subject were also excluded. Exemption of the informed consent requirement for the usage of residual CSF specimens was obtained at each study site in compliance with the local regulations and/or local ethics committee (EC) requirements. The collected CSF specimens were de-identified and labeled with a unique identifier. Frozen retrospective specimens kept at −80°C were thawed at 2°C to 8°C and tested within 90 min post-thaw. Specimens were tested on both the QIAstat-Dx platform and the comparator method BioFire FilmArray ME Panel. Following testing, specimens were kept at −70°C or −20°C and kept frozen until further analysis if needed.

### Preparation of contrived samples

Based on global prevalence numbers, a minimum number of clinical specimens was determined for each pathogen. When this minimum number was not obtained via clinical specimens, samples were prepared through a contriving process using negative clinical CSF and commercial strains in order to mimic clinical samples, with the goal of obtaining sufficient performance data. The contrived samples were prepared for six pathogens: enterovirus, HPeV, *M. pneumoniae*, *N. meningitidis*, *S. agalactiae*, and *S. pyogenes*. Information on the strains used, suppliers, and lot numbers are shown in Table S1. All contrived samples were prepared and validated by QIAGEN Research and Development, Manchester, UK. These pathogens were singly spiked into true-negative (TN) clinical cerebrospinal fluid (cCSF), screened, blinded, and shipped to the three clinical testing sites (Herlev, Denmark; Berlin, Germany; La Tronche, France). See Supplemental Methods for details on cCSF and contrived samples screening.

### Testing with the QIAstat-Dx ME Panel

The QIAstat-Dx ME Panel is a closed-system platform with a panel that identifies microbial agents for meningitis and/or encephalitis. Testing of CSF specimens and analysis of results were carried out according to the manufacturer’s instructions (11). Operators who tested clinical specimens were blinded to SoC results of the specimens. A total of 200 µL CSF was loaded into a QIAstat-Dx ME Panel cartridge, which contained reagents for cell lysis and nucleic acid isolation and amplification. Each QIAstat-Dx ME Panel cartridge encloses an internal control—a titered yeast, *Schizosaccharomyces pombe*.

If the internal control failed to amplify, all negative results were invalid but positive results for detected pathogens were considered valid. Results were obtained in around 79 min and for each target that was amplified, information on the amplification curve, endpoint florescence, and Ct values were reported. The ME panel was also tested daily with external controls comprising four different positive control mixes of pathogens and a negative control mix (see Supplemental Methods for details).

### Comparator testing

The BioFire FilmArray ME Panel identifies *E. coli* K1, *H. influenzae*, *L. monocytogenes*, *N. meningitidis* (encapsulated), *S. agalactiae*, *S. pneumoniae*, cytomegalovirus, enterovirus, HSV-1, HSV- 6, HHV- 6, HPeV, VZV, and *C. neoformans*/*gattii*. All CSF specimens were tested according to the manufacturer’s instructions (12).

### Discrepant analysis

Testing with QIAstat-Dx ME Panel resulted in a true positive (TP) or TN when concordance was demonstrated with the comparator method (BioFire FilmArray ME Panel). When discordant results arose between QIAstat-Dx ME Panel and the BioFire FilmArray ME Panel—i.e., leading to a false-positive (FP) or false-negative (FN) result—discordant analysis was performed. Samples with discordant results and sufficient volume were tested with molecular methods other than BioFire FilmArray ME platform when these methods were available at the sites. When samples lacked sufficient volume for further analysis, the initial SoC result was considered. Additional SoC methods included PCR, bacterial culture, sequencing, and/or antigen screening (Table S3).

### Statistical analysis

The total number of TP, TN, FP, and FN results were determined for all the samples tested with the QIAstat-Dx Panel compared to the results obtained with the comparator method. Positive percent agreement (PPA) and negative percent agreement (NPA) of the system are analyzed using the BioFire FilmArray results as the reference. PPA is calculated as TP/(TP + FN); NPA is calculated as TN/(TN + FP). PPA and NPA are both reported as a proportion and percentage along with the corresponding binomial two-sided 95% confidence limits, determined using the Wilson score method (13).

## RESULTS

### Sample enrollment

**Fig 1:**
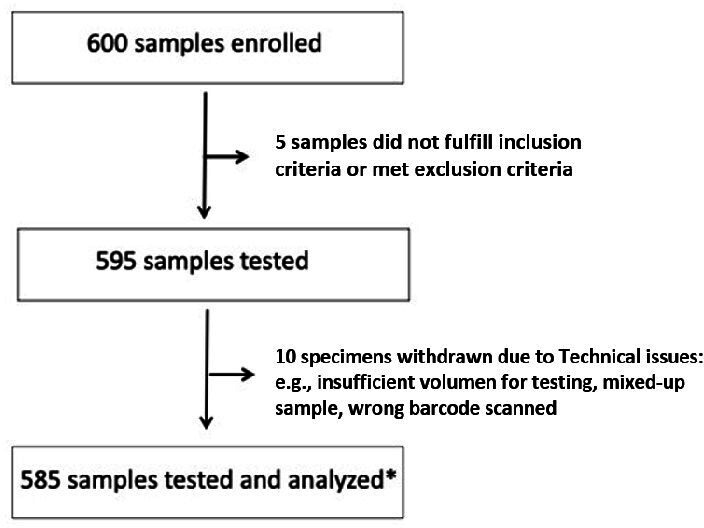
Flow chart diagram depicting the number of samples enrolled and analyzed. Asterisk (*) means that out of the 585 clinical specimens tested, the internal control failed to amplify for 6 specimens, but each of these 6 specimens was found to be positive for a pathogen. The positive results for these six specimens were valid, but the negative results were invalid and were not used in total analysis.

A total of 600 residual retrospective specimens were enrolled in the study. However, a total of 15 samples were excluded from the final analysis due to not fulfilling inclusion criteria or technical issues such as insufficient volume for testing (Fig. 1), resulting in the analysis of the 585 samples.

Demographic information about the patients from which a clinical specimen was included in this study is presented in Table 1. Given that the specimens analyzed were residual and retrospective in nature, demographic information was not available for all the specimens used in the investigation.

**TABLE 1:** Demographics for sample type, gender, and age group of patient cerebrospinal fluid samples tested on the QIAstat-Dx ME Panel platform^*a*^

Variable	Subgroup	*n* (%)
Age group	<2 yr	9 (1.60)
	2–17 yr	24 (4.26)
	18–64 yr	319 (56.56)
	≥65 yr	212 (37.59)
Gender	Female	282 (50.00)
	Male	282 (50.00)

^*a*^ Demographics for 21 clinical samples were not available.

### QIAstat-Dx ME Panel findings and test performance

#### Clinical specimens

In this investigation, 7,533 total targets were tested in clinical samples, resulting in 99.7% (7,512/7,533) agreement between the QIAstat-Dx ME Panel and the BioFire FilmArray ME Panel. The most frequently detected microorganisms were VZV, HSV-2, HSV-1, and *S. pneumoniae*, which were detected in 52 (8.89%), 21 (3.59%), 20 (3.42%), and 15 (2.56%) specimens, respectively (Table 2). Three targets—*S*. *pyogenes*, *M. pneumoniae*, and HPeV—were not detected in any of the clinical samples tested.

**TABLE 2:** PPA and NPA of QIAstat-Dx ME Panel performance by target based on concordance with results from BioFire FilmArray ME Panel^*a*^

Pathogen type	Pathogen	PPA		NPA	
		TP/(TP + FN)	% (95% CI)	TN/(TN + FP)	% (95% CI)
All	All pathogens	135/146	92.5 (87.0–95.7)	7,377/7,387	99.9 (99.8–99.9)
Bacteria	*Escherichia coli* K1	1/1	100.0 (20.7–100.0)	579/579	100.0 (99.3–100.0)
	*Haemophilus influenzae*	4/5	80.0 (37.6–96.4)	572/574	99.7 (98.7–99.9)
	*Listeria monocytogenes*	1/1	100.0 (20.7–100.0)	578/578	100.0 (99.3–100.0)
	*Neisseria meningitidis* (encapsulated)	1/1	100.0 (20.7–100.0)	578/578	100.0 (99.3–100.0)
	*Streptococcus agalactiae*	3/3	100.0 (43.9–100.0)	576/576	100.0 (99.3–100.0)
	*Streptococcus pneumoniae*	15/17	88.2 (65.7–96.7)	561/562	99.8 (99.0–100.0)
	Overall (bacteria)	25/28	89.3 (72.8–96.3)	3,444/3,447	99.9 (99.7–100.0)
Virus	Enterovirus	7/9	77.8 (45.3–93.7)	569/570	99.8 (99.0–100.0)
	Herpes simplex virus 1	20/20	100.0 (83.9–100.0)	561/561	100.0 (99,3–100.0)
	Herpes simplex virus 2	21/23	91.3 (73.2–97.6)	555/557	99.6 (98.7–99.9)
	Human parechovirus			579/579	100.0 (99.3–100.0)
	Human herpes virus 6	9/10	90.0 (59.6–98.2)	568/570	99.7 (98.7–99.9)
	Varicella zoster virus	52/55	94.6 (85.2–98.1)	523/525	99.6 (98.6–99.9)
	Overall (virus)	109/117	93.2 (87.1–96.5)	3,355/3,362	99.8 (99.6–99.9)
Yeast	*Cryptococcus neoformans*/*gattii* (not differentiated)	1/1	100.0 (20.7–100.0)	578/578	100.0 (99.3–100.0)

^*a*^ PPA analysis includes only targets with reference results detected in clinical samples tested, thus *Mycoplasma pneumoniae*, *Streptococcus pyogenes*, and human parechovirus are excluded. Specificity analysis includes targets with reference results not detected in clinical samples. *Mycoplasma pneumoniae* and *Streptococcus pyogenes* are excluded because they were not detected in the clinical samples tested and they are not tested by the BioFire FilmArray ME Panel. In this study, the internal control failed to amplify for six clinical specimens. However, these specimens tested positive for the following pathogens: *Escherichia coli* K1 (one case), herpes simplex virus 1 (two cases), herpes simplex virus 2 (one case), varicella zoster virus (one case), and human herpes virus 6 (one case). These positive results were considered valid, while the rest of the results for that run were considered invalid.

Overall, there was 92.5% (135/146) PPA (Table 2) for all targets detected and 99.9% (7,377/7,387) NPA between the QIAstat-Dx ME Panel and BioFire FilmArray ME Panel (Table 2). The PPA values were ≥90.0% for each of the targets detected, except for *H. influenzae* (80.0%; 4/5), *S. pneumoniae* (88.2%; 15/17), and enterovirus (77.8%; 7/9) (Table 2). Six analytes showed a PPA of 100.0%: *E. coli* K1 (1/1), *L. monocytogenes* (1/1), *N. meningitidis* (encapsulated) (1/1), *S. agalactiae* (3/3), HSV-1 (20/20), and *C. neoformans*/*gattii* (1/1). Three targets demonstrated PPA values between 90.0% and 95.0%: HSV-2 (91.3%; 21/23), HHV-6 (90.0%; 9/10), and VZV (94.6%; 52/55).

#### Testing of contrived samples

All bacterial specimens tested on the QIAstat-Dx ME Panel at concentrations of 0.63× limit of detection (LoD) and 3.16× LoD demonstrated a 100.00% detection rate, with only one exception. The *S. agalactiae* contrived specimens at a concentration of 3.16× LoD exhibited a 96.00% detection rate (24/25) (Table 3). Additionally, the contrived samples for *N. meningitidis* were incorrectly manufactured at a higher concentration than intended and tested at 6.33× LoD and 31.6× LoD, with all samples successfully detected. For viruses, the HPeV and enterovirus specimens tested at 3.16× LoD showed a detection rate of 100.00% (59/59) and 96.67% (58/60), respectively (Table 3).

**TABLE 3:** Contrived sample testing results for each target and concentration^*a*^

Grouping variable(s)			Proportion of detected samples out of total samples		Two-sided 95% confidence limit	
Pathogen type	Target (expected)	Concentration	Fraction	%	Lower	Upper
Bacteria	*Mycoplasma pneumoniae*	3.16× LoD	26/26	100.00	87.13	100.00
		0.63× LoD	35/35	100.00	90.11	100.00
	*Neisseria meningitidis*	6.33× LoD	27/27	100.00	87.54	100.00
		31.6× LoD	38/38	100.00	90.82	100.00
	*Streptococcus agalactiae*	3.16× LoD	24/25	96.00	80.46	99.29
		0.63× LoD	36/36	100.00	90.36	100.00
	*Streptococcus pyogenes*	3.16× LoD	25/25	100.00	86.68	100.00
		0.63× LoD	36/36	100.00	90.36	100.00
Virus	Enterovirus	3.16× LoD	58/60	96.67	88.64	99.08
	Human parechovirus	3.16× LoD	59/59	100.00	93.89	100.00

^*a*^ Contrived samples were considered valid if the correct pathogen was detected using the QIAstat-Dx ME Panel assay.

### Discrepant analysis

Discordance analysis revealed a total of 21 instances of discrepancies between QIAstat-Dx ME Panel and BioFire FilmArray ME Panel, with 10 FP and 11 FN detections. In cases where the original SoC method was specific to the target in question, and the method was not the BioFire FilmArray ME Panel, the original SoC result was used to resolve the discordance. When the original SoC method was FilmArray and sufficient volume remained, discrepant resolution testing was performed with an alternative method to resolve these discrepancies. Of the 21 samples exhibiting discordance, seven samples were not subjected to additional testing due to insufficient volume. For these seven samples, discordance was resolved by referring back to the initial SoC test results (Table S3).

Retesting analysis showed that there was supporting evidence for five FP cases and four FN cases for the QIAstat-Dx ME Panel results (Table 4; Table S4). The five resolved FP cases confirmed to be TP were for *S. pneumoniae* (one case), enterovirus (one case), HHV-6 (one case), and HSV-2 (two cases). The four resolved FN cases confirmed to be TN were for enterovirus (one case), *S. pneumoniae* (two cases), and *H. influenzae* (one case).

**TABLE 4:** Analysis of discordant test results for QIAstat-Dx ME Panel

	False-negative results			False-positive results		
Analyte	Total (*n*)	True negative confirmed (*n*)	False negative confirmed (*n*)	Total (*n*)	True positive confirmed (*n*)	False positive confirmed (*n*)
*Haemophilus influenzae*	1	1	0	2	0	2
*Streptococcus pneumoniae*	2	2	0	1	1	0
Enterovirus	2	1	1	1	1	0
Human herpes virus 6	1	0	1	2	1	1
Herpes simplex virus 2	2	0	2	2	2	0
Varicella zoster virus	3	0	3	2	0	2
Total (all analytes)	11	4	7	10	5	5

QIAstat-Dx ME Panel testing indicated codetections of more than one pathogen in four clinical samples, each had two pathogens identified. Discordance analysis ruled out the presence of coinfection in three of these samples; the fourth sample was confirmed to be co-infected with enterovirus and HHV-6.

## DISCUSSION

Large multiplex PCR panels offer multiple advantages over standard PCR methods for diagnosing ME. In addition to the capacity to rapidly detect several different microorganisms that can cause ME, the sample preparation process is simple, quick, and easy to master, with no precision pipetting required. Unlike standard PCR, which is typically performed only once or twice daily, samples that arrived in the laboratory can be analyzed on large cassette-based panels such as QIAstat-ME Panel immediately upon arrival in the laboratory and may help to perform those analyses on a 24/7 basis.

In this report, we assessed the performance of QIAstat-Dx ME Panel in comparison to the BioFire FilmArray ME Panel. Overall, the QIAstat-Dx ME Panel demonstrated comparable performance with the BioFire FilmArray ME Panel. After discordance analysis, the overall PPA and NPA for all targets detected in clinical samples for QIAstat-Dx ME Panel were 95.2% (140/147) and 99.9% (7,381/7,386), respectively. Most of the bacterial targets identified in the clinical samples were *S. pneumoniae* (16 samples), followed by *H. influenzae* (four samples) and *S. agalactiae* (three samples), which are representative of bacterial meningitis epidemiology in Europe and the United States (8). Viral pathogens were detected in a total of 113 samples, with VZV being the most common (52 samples), followed by HSV-2 (23 samples) and HSV-1 (20 samples). HHV-6 was identified in 10 samples, and enterovirus was detected in 8 samples. HSV and VZV have been identified as the most frequent pathogen and second-most-frequent pathogen, respectively, that cause viral encephalitis in Europe (14).

In 2015, the BioFire FilmArray ME Panel (bioMérieux, France) was the first ME panel to be FDA approved. This panel can detect 6 bacterial, 7 viral, and 1 fungal target in CSF clinical specimens, displaying PPA of 100.0% for 9 targets and NPA of >99.0% for all 14 targets using conventional assays such as bacterial culture, PCR singleplex assays, and DNA sequencing as comparator methods in a multicenter evaluation (9). However, there were 22 unresolved FP and 10 unresolved FN detections in that study, possibly due to specimen contamination during the testing process (9). Since the introduction of the BioFire FilmArray ME Panel, there have been reports cautioning users of low sensitivity for some targets with this system, in particular HSV-1 (15) and *Cryptococcus neformans*/*gattii* (16, 17). In this study, there were 20 HSV-1 and 1 *Cryptococcus* samples detected equally by both platforms, leading to the belief that the same sensitivity issues could be expected for QIAstat-Dx Meningitis/Encephalitis Panel. It should be noted that guidelines for the diagnosis and treatment of encephalitis recommend that in patients with a negative PCR for HSV, consideration should be given to repeating the test 3–7 days later in those with a compatible clinical syndrome or temporal lobe lesions on neuroimages (4). The most sensitive diagnostic test for cryptococcal meningitis remains CrAg (cryptococcal antigen) but, as this test can remain positive long after the elimination of the pathogen from the patient, the definitive diagnosis is made by culture from the CSF, which can take up to 7 days to grow (18). Results from multiplex panels should always be interpreted in the context of each patient, and final diagnosis be made considering all available test results as well as clinical presentation.

In this study, confirmed FP detections could possibly be attributed to contamination occurring during the workflow process (e.g., while pipetting and transferring samples) (19 – 21). Thus, preventive measures, such as cleaning the laboratory bench after every sample, are essential. The majority of FN detections observed in this study were for herpesviruses: VZV (three samples), HSV-2 (two samples), and HHV-6 (one sample). A possible cause of FN detection is a pathogen load below the LoD of the assay (13).

Multiplex PCR assays such as the QIAstat-Dx ME Panel are also able to identify coinfections. In this study, a coinfection of HHV-6 and enterovirus was detected in one sample. Although coinfections are rare, and their clinical significance in CSF samples is poorly understood, other groups have also reported the detection of coinfections. For instance, in a study evaluating the performance of the BioFire FilmArray ME Panel, Liesman and colleagues also detected and confirmed 3 samples with coinfections among a total of 291 samples analyzed (22). The QIAstat-Dx ME Panel can detect two additional bacterial targets that are not detected by the BioFire FilmArray ME Panel: *M. pneumoniae* and *S. pyogenes*, but none were detected during the clinical study. *M. pneumoniae* is reportedly one of the major causative agents of encephalitis in children—between 5% and 10% of these cases are caused by this pathogen (23). Invasive infections of *S. pyogenes* resulting in meningitis are rare (1%–2%), but these infections have a high mortality rate (23%–27%) (24). A unique feature of the QIAstat-Dx ME testing platform is that it provides amplification curve, endpoint fluorescence, and Ct information for all targets. This latter parameter is an indicator of the strength or weakness of a positive signal and, consequently, of the pathogen load in the CSF, which may help clinicians in the management of the patient.

This study has several limitations. Due to the limited availability of residual CSF samples, the study was carried out with retrospective samples. In this investigation, 29 contrived samples required repeat testing, either due to failure of internal control (22 samples) or failure of test status (7 samples). Due to the difficulty to obtain high volumes of pathogen-free clinical CSF, repeat tests were not able to be performed, and only samples that yielded valid positive or negative results were considered in the analysis. For the pathogens not present in the BioFire FilmArray ME Panel, not enough volume was available to test with a different comparator method, which could have potentially led to unidentified FN results for *M. pneumoniae* and *S. pyogenes*. For *Cryptococcus*, despite it being a relatively common cause of meningoencephalitis, only one clinical sample was tested, and there was no contriving done. Further evaluation is needed to better understand the performance of QIAstat-Dx ME Panel for this pathogen.

In summary, the QIAstat-Dx ME Panel exhibited similar performance relative to the comparator method—the BioFire FilmArray ME Panel—in this clinical trial study involving three testing sites in Europe. The results obtained in this clinical performance study contribute to the body of evidence demonstrating the scientific validity of multiplex PCR panels in detecting and identifying bacterial, viral, and fungal pathogens to aid in diagnosing specific causative agents of meningitis and/or encephalitis, in conjunction with SoC techniques, such as culture for organism recovery, serotyping, genotyping, and antimicrobial susceptibility testing. Ultimately, multiplex PCR will likely replace some SoC methods, and the QIAstat-Dx ME Panel offers a valuable new alternative for multiplex PCR testing for ME. While the QIAstat-Dx ME Panel is a qualitative assay, it is unique in that—unlike the BioFire FilmArray ME Panel—it also provides information on Ct values for its targets, which reflects a sample’s pathogenic load; this information may be useful in guiding physicians on patient management and adds a layer of data that might help interpret results in the laboratory. Featuring a robust and accurate assay, the panel facilitates rapid and comprehensive testing for community-acquired meningoencephalitis.

## SUPPLEMENTAL MATERIAL

Additional experimental details jcm.00426-23-s0001.docx Supplemental methods and supplemental tables (S1 through S4).

ASM does not own the copyrights to Supplemental Material that may be linked to, or accessed through, an article. The authors have granted ASM a non-exclusive, world-wide license to publish the Supplemental Material files. Please contact the corresponding author directly for reuse.
