# Supplementary material for: Evaluation of the QIAstat-Dx Meningitis/Encephalitis Panel, a multiplex PCR platform for the detection of community-acquired meningoencephalitis
Source: J Clin Microbiol. 2023 Sep 13;61(10):e00426-23. doi: 10.1128/jcm.00426-23 (PMC10595057; doi:10.1128/jcm.00426-23)
Supplement: Additional experimental details — Supplemental methods and supplemental tables (S1 through S4). [file jcm.00426-23-s0001.docx]

**SUPPLEMENTAL METHODS**

**Preparation of contrived samples**

*cCSF screening*

Prior to spiking the cCSF with pathogens, the cCSF (BIOMEX) utilized in the preparation of the contrived samples were screened to confirm that it was negative for the 15 pathogens detected by QIAstat-Dx ME Panel. This was performed by testing 3 replicates of the cCSF samples on each of the following panels: Meningitis/Encephalitis FilmArray Panel (BioFire), Pneumonia Plus FilmArray Panel (BioFire), and QIAstat-Dx ME Panel. All cCSF samples were determined to be negative upon testing on each of the 3 multiplex PCR panels.

*Screening of contrived samples*

Each pathogen was spiked into cCSF to 2 final concentrations as shown in Table S2. The contrived samples were aliquoted into vials with each vial containing 220 µl and stored below -60°C prior to screening.

**Testing with the QIAstat-Dx ME Panel: External controls**

A total of 4 different external control mixes, prepared in artificial CSF media, were also tested at each site. Mix 1 was composed of *S. pneumoniae, N. meningitidis* (encapsulated), *S. agalactiae, L. monocytogenes*, *H. influenzae*, and enterovirus A/B/C; Mix 2 of HSV-1, HHV-6, VZV, and *S. pyogenes*; Mix 3 of *M. pneumoniae*, *C. gattii*, HPeV, HSV-2, and *E. coli* K1; and Mix 4 was negative (no pathogens). Each day during testing, 1 positive external control mix and the negative control mix were analyzed. Each of the 3 positive external controls was tested in a rotating manner. Thus, over the course of 3 consecutive days all 3 positive external control mixes were run. If a testing site utilized more than 1 QIAstat-Dx analytical module for the investigation, testing with the positive external control and negative external control were conducted on alternating analytical modules each day of testing. The results of both the positive and negative external control mixes had to be valid (correct) for the results of the clinical and contrived specimens to be valid on any given testing day.

**SUPPLEMENTAL TABLES**

**Table S1.** Information of pathogens used to prepare contrived samples

| **Pathogen** | **Strain** | **Catalog ID** | **Supplier** | **Lot number** |
| --- | --- | --- | --- | --- |
| Enterovirus | EV 70, species D, strain J670/71 | VR-836 | ATCC | 70010613 |
| Human parechovirus | Serotype 3 | 0810147CF | ZeptoMetrix | 321353 (sublot: 532063) |
| *Mycoplasma pneumoniae* | PI 1428 | 29085 | ATCC | 70021660 |
| *Neisseria meningitidis* | Serotype B. M2092 CIP 104218 L | 13090 | ATCC | 70023133 |
| *Streptococcus agalactiae* | G19 group B | 13813 | ATCC | 70011789 |
| *Streptococcus pyogenes* | Z472; Serotype M1 | 0804351 | ZeptoMetrix | 324707 |

**Table S2**. LoD values for the pathogens which were used to prepare contrived samples

|  | | | **Concentration of the contrived samples** | |
| --- | --- | --- | --- | --- |
| **Pathogen** | **1× LoD** | **Units** | **High concentration (× LoD)** | **Low concentration (× LoD)** |
| Enterovirus | 5.00E+00 | TCID50/mL | 3.16 | 0.63 |
| Human parechovirus | 1.07E+01 | TCID50/mL | 3.16 | 0.63 |
| *Mycoplasma pneumoniae* | 3.00E+01 | CFU/ml | 3.16 | 0.63 |
| *Neisseria meningitidis* | 2.62E-01 | CFU/ml | 31.64 | 6.33 |
| *Streptococcus agalactiae* | 1.07E+03 | CFU/ml | 3.16 | 0.63 |
| *Streptococcus pyogenes* | 5.69E+02 | CFU/ml | 3.16 | 0.63 |

**Table S3**. Standard of care and reference methods used for the detection of each pathogen

| **Targets** | **Standard of Care (varied across all sites)** | **Study** | **Reference method** | **Discordance resolution** |
| --- | --- | --- | --- | --- |
| *Escherichia coli* K1 | FilmArray and PCR | QIAstat-Dx ME Panel | BioFire® FilmArray® ME panel | SoC or Validated endpoint PCR |
| *Haemophilus influenzae* | FilmArray |  |  |  |
| *Listeria monocytogenes* | Culture and FilmArray |  |  |  |
| *Neisseria meningitidis* (encapsulated) | FilmArray |  |  |  |
| *Streptococcus agalactiae* | FilmArray |  |  |  |
| *Streptococcus pneumoniae* | Culture, FilmArray, PCR |  |  |  |
| *Streptococcus pyogenes* | N/A |  | Validated endpoint PCR |  |
| *Mycoplasma pneumoniae* | N/A |  | Validated endpoint PCR |  |
| Herpes simplex virus 1 | PCR and FilmArray |  | BioFire® FilmArray® ME panel |  |
| Herpes simplex virus 2 | PCR and FilmArray |  |  |  |
| Human herpes virus 6 | PCR and FilmArray |  |  |  |
| Enterovirus | PCR and FilmArray |  |  |  |
| Parechovirus | N/A |  |  |  |
| Varicella zoster virus | PCR, sequencing, FilmArray |  |  |  |
| *Cryptococcus neoformans/gattii* (not differentiated) | FilmArray, antigen screening, culture |  |  |  |

N/A, not available

**Table S4.** Resolution of individual discordant results for BioFire® FilmArray® versus QIAstat-Dx ME Panel

|  |  | **Initial analysis** | | | **Discrepant analysis** | | | |
| --- | --- | --- | --- | --- | --- | --- | --- | --- |
| **Subject^1^** | **Target^2^** | **BioFire FilmArray** | **QIAstat-Dx-ME** | **Result^3^** | **SoC test method** | **SoC result** | **SoC**  **Pathogen** | **Final outcome** |
| 1 | Herpes simplex virus 2 | Negative | Positive | FP | PCR | Positive | Herpes simplex virus 2 | TP |
| 2 | Human herpes virus 6 | Negative | Positive | FP | PCR | Positive | Varicella zoster virus | FP ^4^ |
| 3 | Herpes simplex virus 2 | Positive | Negative | FN | PCR | Positive | Herpes simplex virus 2 | FN |
| 4 | Enterovirus | Positive | Negative | FN | BioFire FilmArray | Positive | Enterovirus | FN |
| 5 | Herpes simplex virus 2 | Negative | Positive | FP | PCR | Positive | Herpes simplex virus 2 | TP |
| 6 | Herpes simplex virus 2 | Positive | Negative | FN | BioFire FilmArray | Positive | Herpes simplex virus 2 | FN |
| 7 | Varicella zoster virus | Positive | Negative | FN | PCR | Positive | Varicella zoster virus | FN |
| 8 | Varicella zoster virus | Positive | Negative | FN | PCR | Positive | Varicella zoster virus | FN |
| 9 | *S. pneumoniae* | Negative | Positive | FP | BioFire FilmArray | Positive | *S.* *pneumoniae* | TP |
| 10 | Enterovirus | Positive | Negative | FN | PCR | Negative | Enterovirus not detected | TN |
| 10 | Varicella zoster virus | Negative | Positive | FP | PCR | See footnote below^6^ | Varicella zoster virus not detected | FP |
| 11 | Varicella zoster virus | Negative | Positive | FP | BioFire FilmArray | Negative | Varicella zoster virus not detected | FP |
| 12 | *H. influenzae* | Negative | Positive | FP | BioFire FilmArray | Positive | Varicella zoster virus | FP**^5^** |
| 13 | Enterovirus | Negative | Positive | FP | PCR | Positive | Enterovirus | TP |
| 14 | Varicella zoster virus | Positive | Negative | FN | PCR | Positive | Varicella zoster virus | FN |
| 15 | *H. influenzae* | Negative | Positive | FP | PCR | Negative | *H. influenzae* not detected | FP |
| 16 | *H. influenzae* | Positive | Negative | FN | PCR | Negative | *H. influenzae* not detected | TN |
| 17 | Human herpes virus 6 (HHV-6) | Positive | Negative | FN | Tested for HHV-6 infection | Positive | Human herpes virus 6 | FN |
| 18 | Human herpes virus 6 | Negative | Positive | FP | PCR | Positive | Human herpes virus 6 | TP |
| 19 | *S. pneumoniae* | Positive | Negative | FN | PCR | Positive | *S. pneumoniae* not detected | TN |
| 20 | *S. pneumoniae* | Positive | Negative | FN | PCR | Positive | *S. pneumoniae* not detected | TN |

FN, false negative; FP, false positive; TN, true negative; TP, true positive

^1^For this discordant analysis, patients were assigned arbitrary numbers to indicate that these are different residual CSF specimens. In certain cases, more than one pathogen was detected in a given sample (Subject no. 10).

^2^“Target” refers to the pathogen initially detected using either BioFire® FilmArray®**,** the comparator method or QIAstat-Dx ME Panel.

^3^Based on the initial analysis, the target tested was designated either a FN or a FP result.

^4^Varicella zoster virus was detected and not human herpes virus 6. This result is a FP with respect to the presence of human herpes virus 6.

^5^The SoC method employed was BioFire® FilmArray® Panel and was found to be positive for varicella zoster virus whereas *Haemophilus influenzae* was not detected. This is a FP with respect to the presence of *Haemophilus influenzae* detected by QIAstat-Dx ME Panel.

^6^ This is an old specimen dating back to 2017 and was subjected to an old workflow. This sample was tested for herpes simplex virus 2 using the QIAGEN Artus HSV-PCR and herpes simplex virus 2 was detected. No other alternative SoC method was available at the designated site.
